# Identification of a particle collision as a finite-time blowup in turbulence

**DOI:** 10.1038/s41598-022-27305-5

**Published:** 2023-01-05

**Authors:** Seulgi Lee, Changhoon Lee

**Affiliations:** 1 Department of Mechanical Engineering, Yonsei University, Seoul, 03722 South Korea; 2 Department of Mechanical Engineering & School of Mathematics and Computing, Yonsei University, Seoul, 03722 South Korea

**Keywords:** Fluid dynamics, Mechanical engineering

## Abstract

We propose an Eulerian approach to investigate the motion of particles in turbulence under the assumption that the motion of particles remains smooth in space and time until a collision between particles occurs. When the first collision happens, particle velocity loses $C^1$ continuity, resulting in a finite-time blowup. The corresponding singularities in particle velocity gradient, particle number density, and particle vorticity for various Stokes numbers and gravity factors are numerically investigated for the first time in a simple two-dimensional Taylor-Green vortex flow, two-dimensional decaying turbulence, and three-dimensional isotropic turbulence. In addition to the critical Stokes number above which a collision begins to occur, the flow condition leading to collision is revealed; particles tend to collide in very thin shear layer constructed by two parallel same-signed vortical structures when Stokes number is above the critical one.

## Introduction

Particle-laden turbulence is frequently observed in daily life and industrial applications such as droplets settling in clouds and precipitation processes, particles sedimenting in river, aerosol pollutants in the air, fuel droplets in a diesel engine, and particles in a chemical reactor. Often, collisions between particles play a critical role in the determination of particle behavior. A good example is rain formation in which the collision rate between settling droplets is critically affected by background turbulence^1–10^.

For decades, the preferential concentration or clustering of inertial particles, which has been identified to be responsible for enhanced collision between particles, has been actively investigated using direct numerical simulation (DNS)^11–19^. The primary mechanism of the preferential concentration in the absence of gravity is the centrifuge effect of the inertial particles around rotating structures of turbulence. It is well characterized by the ratio of the particle response time scale $\tau _p$ to the fluid time scale $\tau _f$, known as the Stokes number $St=\tau _p/\tau _f$. Especially when $St\sim 1$, preferential concentration is most pronounced in regions where fluid rotation is weak, but straining motion is not. The interaction between laden particles and turbulent coherent structures preferentially determines the accumulation pattern^20,21^. Moreover, when the settling motion of inertial particles due to gravity is dominant, a new type of preferential accumulation in columnar structures was observed^10,19,22,23^. Such a clustering naturally leads to more collision between particles. Although numerous studies on particle clustering revealed the physical mechanism and provided clues on how collision modeling can be improved, a full understanding of physical mechanism of collision lacks. Reference^24^ attempted to investigate the collision mechanism tracking colliding finite-sized particles in direct numerical simulation of particle-laden isotropic turbulence. They showed that depending on Stokes number colliding pattern varies. However, an investigation of a collision event in the Lagrangian simulation of particles has limitation since it requires a luck to have a collision and thus it needs a large number of particles to be tracked to increase the chance of collision.

Equations of particle motion considered in this study are given by

1 $$\begin{aligned} \frac{d\varvec{r}}{dt}=\varvec{v},~~ \frac{d \varvec{v}}{dt}= \frac{\varvec{u}\left( \varvec{r},t\right) -\varvec{v}}{\tau _p}+\varvec{g} , \end{aligned}$$

where $\varvec{r}$ and $\varvec{v}$ are the position and velocity of a particle, respectively. $\varvec{u}$ is fluid velocity at the particle’s position, which is a solution of Navier–Stokes equation and $\varvec{g}$ is the gravitational acceleration vector. $\tau _p$ is called the relaxation time of a small spherical particle defined by $\tau _p=2\rho _p a^2/9\rho \nu$ with $\rho _p$, $\rho$, $\nu$ and *a* denoting particle density, fluid density, fluid viscosity, and the particle radius. Equation (1) is valid under the point-particle approximation that the particle size is much smaller than the smallest flow length scale such as the Kolmogorov length scale when the density ratio of a particle relative to fluid is much larger than 1 so that all other forces such as the Basset history forces, the added mass and the fluid acceleration are negligible. It should be noted that when the density ratio is very large such as an order of 1000 (water droplet in the air), a small particle of order of 10 microns in cloud environment easily results in Stokes number of order one or larger.

An inspiring characterization of particle collision was proposed by ref.^1^ some time ago, who derived a formula for the collision rate of small inertial particles in turbulence using a form that traces random trajectories^25,26^. They introduced a *particle velocity field* $\varvec{v}(\varvec{x},t)$ from a solution for small Stokes number of equations of particle motion. Particle velocity field $\varvec{v}( \varvec{x},t)$ defining the velocity of a particle located at $\varvec{x}$ at time *t* satisfies the equation $\partial _t \varvec{v} +(\varvec{v} \cdot \nabla )\varvec{v}=(\varvec{u}-\varvec{v})/\tau _p + \varvec{g}$, which is equivalent to Eq. (1) under the assumption that the particle velocity field is a smooth function of space. Taking gradient of this equation yields, in a qualitative form, $\partial _t{\sigma }+(\varvec{v} \cdot \nabla ) \sigma +\sigma ^2 = (s-\sigma )/\tau _p$ where $\sigma =\nabla \varvec{v}$ and $s=\nabla \varvec{u}$. (This is made more precise in the next section.) When $|\sigma | \ll \tau _p^{-1}$, it has a smooth evolution determined by $\sigma (t) = \int ^{t}dt' \exp [(t'-t)/\tau _p]s(t')/\tau _p$. If $|\sigma | > \tau _p^{-1}$, however, $\sigma ^2$ dominates and it may lead to an explosive evolution $\sigma (t) \propto (t_0-t)^{-1}$ that produces singularity in $\varvec{v}(\varvec{x},t)$ in finite time. Although this estimate is based on the order-of-magnitude analysis, the singularity of the velocity gradient of a particle implies that the particle velocity develops blowup and becomes multivalued; in other word, a particle can have two different velocities at the same position, implying collision between two particles. Although compressibility of particle velocity or particle number density has been numerically investigated in turbulence using the Lagrangian method^6,27–29^ and the Eulerian model^30^, a clear identification of collision has not been carried out. Therefore, we propose a more rigorous Eulerian approach in which the particle velocity field $\varvec{v}(\varvec{x},t)$ is directly handled for an investigation of collision process of particles.

## Results

### Eulerian simulation of particle motion

We introduce a direct Eulerian simulation of particle motion within finite time. Equation (1) allows the following solution for the particle velocity,

2 $$\begin{aligned} \varvec{v}\left( t\right) = \varvec{w} + \frac{1}{\tau _p} \int _{-\infty }^{t} \exp \left( \frac{t'-t}{\tau _p} \right) \varvec{u} \left( \varvec{r}\left( t'\right) ,t'\right) dt', \end{aligned}$$

where $\varvec{w}=\varvec{g}\tau _p$ is the settling velocity of a particle in still fluid. The particle velocity is determined by accumulated information of fluid velocity along a particle trajectory, which prevents further explicit expression. When $\tau _p$ is small, i.e., a particle is small or light, however, the following approximation can be derived using the Taylor expansion of $\varvec{u} (\varvec{r} (t'),t')$ for small $t'-t$,

3 $$\begin{aligned} \!\!\!\varvec{v}(t) \simeq \varvec{w} + \varvec{u}\left( \varvec{r}(t),t\right) - \tau _p \left( \frac{\partial \varvec{u}}{\partial t} + ((\varvec{u}+\varvec{w}) \cdot \nabla )\varvec{u}\right) . \end{aligned}$$

Given that terms on the right-hand side of Eq. (3) are smooth functions of space, the particle velocity is uniquely determined by the position. Hence, the so-called *flow of particle* $\varvec{v}(\varvec{x},t)$ exists^10^. However, when $\tau _p$ is not small, such an approximation is not possible because of the nonlocal nature of the solution, Eq. (2). As far as the particle velocity $\varvec{v}(\varvec{x},t)$ remains smooth, Eq. (1) can be written in the Eulerian form as

4 $$\begin{aligned} \frac{\partial \varvec{v}}{\partial t} + \left( \varvec{v}\cdot \nabla \right) \varvec{v} = \frac{\varvec{u}-\varvec{v}}{\tau _p} + \varvec{g}. \end{aligned}$$

Even when $\tau _p$ is not small, Eq. (4) is valid under the condition that the particle velocity remains smooth because the left hand side of Eq. (4) corresponds simply to the Taylor expansion of the smooth particle velocity along a particle path. As will be shown later, the particle velocity remains smooth for a while when it was initially smooth until a blowup occurs. Then, the asymptotic behavior of the particle velocity leading to a blowup can be investigated using Eq. (4).

Taking the gradient of Eq. (4) yields an equation for $\sigma _{ij} (=\partial v_i/\partial x_j)$,

5 $$\begin{aligned} \frac{D\sigma _{ij}}{Dt} + \sigma _{ik}\sigma _{kj} = \frac{s_{ij}-\sigma _{ij}}{\tau _p}, \end{aligned}$$

where $D/Dt (=\partial /\partial t + \varvec{v} \cdot \nabla )$ denotes the material derivative along a particle path and $s_{ij}=\partial u_i/\partial x_j$. In the order-of-magnitude sense^10^, Eq. (5) reduces to

6 $$\begin{aligned} \frac{D\sigma }{Dt} + \sigma ^2 = \frac{s-\sigma }{\tau _p}, \end{aligned}$$

where $\sigma$ and *s* denote scalar variables representing $\sigma _{ij}$ and $s_{ij}$, respectively. When $\tau _p$ is small such that $\sigma /\tau _p \gg \sigma ^2$, the quadratic term is small, yielding

7 $$\begin{aligned} \sigma \simeq s -\tau _p \left( \frac{D\sigma }{Dt}+\sigma ^2 \right) \simeq s -\tau _p \left( \frac{Ds}{Dt}+s^2 \right) , \end{aligned}$$

which remains finite since *s* is finite.

When $\tau _p$ is large such that $\sigma /\tau _p \ll \sigma ^2$, however, the right-hand side of Eq. (6) is much smaller than the left-hand side, yielding a solution that might blow up in finite time,

8 $$\begin{aligned} \sigma (t) \simeq \frac{1}{t-t_c}. \end{aligned}$$

This kind of behavior leading to a blowup of the gradient of a solution is found in a solution to the inviscid Burgers equation when a shock develops^31^.

Reference^10^ extended this analysis to further show that even when $\tau _p$ is large, the finite-time blowup can be suppressed when gravity is strong or a nondimensional parameter called the Froude number defined by $Fr \equiv \eta /(g \tau _\eta ^2 )$ is much smaller than one. Here, $\eta$ and $\tau _\eta$ are the Kolmogorov length and time scales. This implies that when gravity is sufficiently strong, the particle velocity may remain smooth. However, all these estimates are qualitative based on the order-of-magnitude analysis.

For a more rigorous investigation, Eq. (5), which is the tensorial form of Eq. (6), should be considered for analysis, indicating that the behavior of a solution cannot be simply estimated since the evolution of $\sigma _{ij}$ depends on the self-interaction between components of $\sigma _{ij}$ in a highly complex manner. It is also possible that quadratic nonlinearity does not always lead to a blowup, owing to the depletion of nonlinearity observed in the solution of the Euler equation^32–34^. Only a full numerical investigation of Eq. (4) can reveal the accurate behavior of a solution including the possibility of a blowup.

### Three kinds of background flow considered

To study a complete description of finite-time blowup of particle motion, we propose a direct simulation of Eulerian equation of particle velocity field, Eq. (4) for the given solution to the Navier–Stokes equation $\varvec{u}(\varvec{x},t)$. In this study, we consider three types of the background flow in a cubic domain as described below.

#### 2D Taylor–Green vortex flow

A two-dimensional exact solution to the Navier–Stokes equation in a periodic domain, $[0, 2\pi ]^2$ is given by

9 $$\begin{aligned} u_1=e^{-2\nu t}\cos x_1 \sin x_2, ~~u_2=-e^{-2\nu t}\sin x_1 \cos x_2, \end{aligned}$$

indicating a slowly decaying vortex flow for small $\nu$.

#### 2D decaying turbulence

Two-dimensional velocity field is given by $\varvec{u}(x_1,x_2,t) = -\varvec{k} {\varvec{\times }} \nabla \psi$ with $\varvec{k}$ denoting a unit vector in $x_3-$direction. The stream function $\psi$ satisfies $\nabla ^2 \psi = - \omega$, where the vorticity $\omega (x_1,x_2,t)$ is obtained from solving

10 $$\begin{aligned} \frac{\partial \omega }{\partial t} + (\varvec{u}\cdot \nabla ) \omega = \nu \nabla ^2 \omega \end{aligned}$$

in a periodic domain, $[0,2\pi ]^2$. The initial enstrophy spectrum is specified by $E_\omega (\kappa ,0) = \frac{a_m}{2} \frac{u_0^2}{\kappa _p} \left( \frac{\kappa }{\kappa _p}\right) ^{2m+1} \exp \left[ -\left( m+\frac{1}{2}\right) \left( \frac{\kappa }{\kappa _p}\right) ^2\right]$, where $\kappa =|\varvec{\kappa }|$ with $\varvec{\kappa }$ denoting the wavenumber vector. *m* and $a_m=(2m+1)^{m+1}/2^m m!$ are the shape parameter and the normalization constant, respectively. We set $u_0=10, m=3, \kappa _p=10$ and the detailed description is found in ref.^35^.

#### 3D isotropic turbulence

In a periodic cubic domain $[0,2\pi ]^3$, the three-dimensional velocity field $\varvec{u}(\varvec{x},t)$ is obtained from solving the continuity and Navier-Stokes equations,

11 $$\begin{aligned} \nabla \cdot \varvec{u}= & {} 0, \end{aligned}$$

12 $$\begin{aligned} \frac{\partial \varvec{u}}{\partial t} +(\varvec{u} \cdot \nabla ) \varvec{u}= & {} - \frac{1}{\rho } \nabla p + \nu \nabla ^2 \varvec{u} + \varvec{f} \end{aligned}$$

where *p* is the pressure, and $\varvec{f}$ is a large-scale random force required to maintain stationary turbulence^15,36^. Parameters of $\varvec{f}$ are chosen such that the Reynolds number based on the Taylor-scale, $Re_\lambda$, is maintained at 14, which is intentionally set to be low to capture the blowup behavior well within the limited resolution. See “Methods” section for details of simulation methods and the definitions of the relevant nondimensional parameters.

### Particle velocity fields near a collision

**Figure 1:**
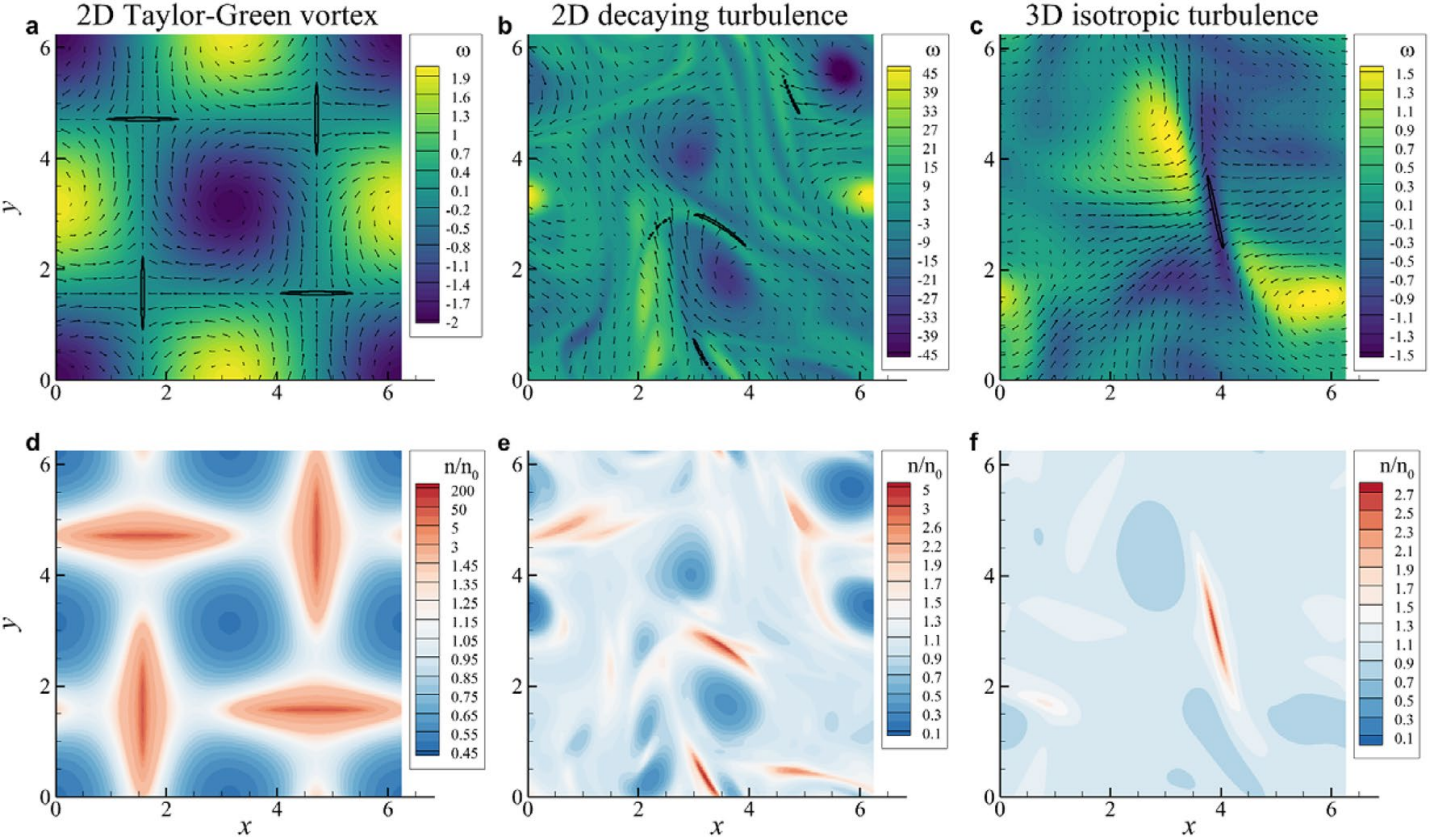
Flow field just before a particle collision. (**a**, **d**) 2D Taylor–Green vortex flow. (**b**, **e**) 2D decaying turbulence. (**c**, **f**) 3D isotropic turbulence. Top panels (**a**–**c**) present the divergence of particle velocity (solid contour), color contours of fluid vorticity $\omega$ and particle velocity vectors. Bottom panels (**d**–**f**) present particle number density normalized by initial particle number density, $n/n_0$. $St=0.8$ and $W=0$ for all cases.

To study the collision process of particles, we introduce a direct Eulerian simulation of particle motion within finite time (Eq. 4). Figure 1 shows the results of flow fields just before a blowup in the three background flows to provide the overall idea of a blowup visually. The region denoted by negatively large value of the divergence of particle velocity (Fig. 1a–c) or locally large value of the particle number density (Fig. 1d–f) corresponds to the place where a blowup is going to occur. As is well-known^10,21,23^, particles with inertia tend to cluster in the region with weak fluid rotation but with strong straining motion. A blowup seems to occur in the region with strong compressive and weak stretching motion marked by a thin long region (Fig. 1), commonly for all three kinds of flow. Indeed, a blowup is easily identified in the Eulerian simulation of particle motion through an investigation of particle velocity field and particle number density distribution, although the simulation is valid only until the first blowup occurs since the simulation blows up numerically as well. In our investigation, we did not consider the size of a particle although the finite size shortens the collision time a little.

### Behavior of particle velocity divergence

A blowup can be identified in the behavior of the divergence of particle velocity (Fig. 1a–c). The evolution equation for the divergence of particle velocity is obtained through taking the trace of the evolution equation of particle velocity gradient (Eq. 5),

13 $$\begin{aligned} \frac{D\sigma _{ii}}{Dt} + \sigma _{ik}\sigma _{ki} = -\frac{\sigma _{ii}}{\tau _p}, \end{aligned}$$

since $s_{ii}=0$ due to incompressibility of fluid flow. The solution to Eq. (13) with the initial condition $\sigma _{ii}(0)=s_{ii}=0$ can be expressed by

14 $$\begin{aligned} \sigma _{ii}(t)= - \int _0^t \exp \left( \frac{t'-t}{\tau _p} \right) \sigma _{ik}\sigma _{ki} (\varvec{r}(t'),t')~dt' , \end{aligned}$$

indicating that the behavior of the quadratic nonlinear term along a particle path is playing an important role in the blowup process of $\sigma _{ii}$. For small $\tau _p$, the integral can be approximated to yield

15 $$\begin{aligned} \sigma _{ii}(t) \simeq -\tau _p \sigma _{ik}(t)\sigma _{ki}(t)=\tau _p (\sigma _{ik}^A \sigma _{ik}^A - \sigma _{ik}^S \sigma _{ik}^S ), \end{aligned}$$

where $\sigma _{ik}^A$ is the skew-symmetric part of $\sigma _{ik}$ called the rotation rate tensor and $\sigma _{ik}^S$ is the symmetric part of $\sigma _{ik}$ called the strain rate tensor. Given that $\sigma _{ik}^A \simeq s_{ik}^A$ and $\sigma _{ik}^S \simeq s_{ik}^S$ for small $\tau _p$, Eq. (15) suggests that an accumulation of particles quantified by negative divergence would occur in the place where the straining motion dominates the rotational motion, but this does not lead to a blowup.

When $\tau _p$ is large, however, the exponential function decays very slowly in the integral of Eq. (14), allowing

16 $$\begin{aligned} \sigma _{ii}(t)\simeq - \int ^t \sigma _{ik}\sigma _{ki} (t')~dt' = -\int ^t \sigma _{ik}^S \sigma _{ik}^S - \sigma _{ik}^A \sigma _{ik}^A ~dt' , \end{aligned}$$

clearly indicating that a blowup could indeed occur through the quadratic nonlinearity of $\sigma _{ik}^S$ since $\sigma _{ii}=tr (\sigma ^S )$ in the particle accumulation process. Furthermore, $\sigma _{ii}=\sum _{j=1}^D \lambda _j$, where *D* is the dimension of space, and $\lambda _j$ is the eigenvalues of $\sigma _{ik}^S$ with $\lambda _1> \cdots > \lambda _D$. Therefore, the most negative eigenvalue $\lambda _D$ would blow up when a blowup of divergence occurs.

**Figure 2:**
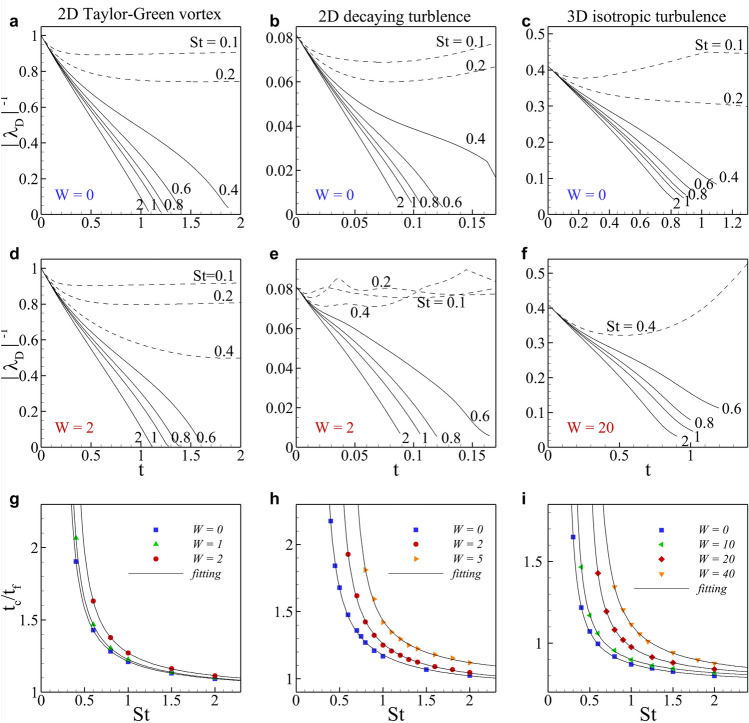
Identification of a particle collision in Eulerian simulation. Eulerian simulation of particle motion used three kinds of background flows such as 2D Taylor–Green vortex flow (**a**, **d**, **g**), 2D decaying turbulence (**b**, **e**, **h**) and 3D isotropic turbulence (**c**, **f**, **i**). (**a**–**f**) Time evolution of maximum absolute negative eigenvalue for various Stokes numbers with gravity. The solid lines denote blowup, and the dashed lines indicate no blowup. Top panels (**a**–**c**) display the results in the absence of gravity, and bottom panels (**d**–**f**) shows the results in the presence of gravity. (**g**–**i**) Critical time as a function of Stokes number *St* according to the gravity parameter *W*. Fitting lines are $t_c/t_f = a/\sinh (St-St_c)+b$.

For an identification of a blowup, the temporal behavior of the $L^\infty$ norm of $|\lambda _D|$ was numerically investigated for the three kinds of flow (Fig. 2a–f). When a blowup (see solid lines in Fig. 2a–f) occurs, $\lambda _D$ exhibits the following behavior commonly for all three kinds of flow,

17 $$\begin{aligned} |\lambda _D| = \frac{C_\lambda }{t_c - t} , \end{aligned}$$

where the critical time $t_c$ is estimated through an extrapolation of $1/|\lambda _D(t)|$. Figure 2a–f clearly confirms that there exists the critical Stokes number $St_c$, above which a blowup occurs, and that the blowup occurs earlier for a larger *St*. When $St > St_c$, implying that the first term on the right-hand side of Eq. (4) is small, a singularity similar to the Burger’s shock naturally forms. When $St < St_c$, however, the first term on the right-hand side of Eq. (4) obviously regularizes the solution by suppressing the divergence of the particle velocity since for small $\tau$ the particle velocity field tends to approach the fluid velocity which is divergence-free.

When gravity is considered and thus particles settle, the blowup is delayed for the same *St*, and even a blowup which occurred in the absence of gravity is not observed (see cases with $St=0.4$ for all three flows in Fig. 2a–f). This is consistent with the theoretical estimation by^10^ in that as gravity becomes stronger, the flow of particles is observed in the wider range of *St*.

The critical time $t_c$ estimated from the behavior of $1/|\lambda _D|$ as a function of *St* for various range of *W* (Fig. 2g–i). For the estimation of $St_c$, a fitting function of $t_c/t_f = a/\sinh (St - St_c)+b$ was adopted. Obtained fitting parameters, $St_c, a$ and *b*, are listed in Table 1. It is noteworthy that $St_c=0.194$ for $W=0$ in 3D isotropic turbulence, while $St_c=0.425$ and 0.511 for $W=20$ and 40, respectively, implying that the first collision between particles could happen at this critical Stokes number, although it might be rare.

**Table 1:** Critical Stokes number at various gravity factors for three kinds of flow. Critical Stokes number is obtained by fitting data (Fig. 2g–i) by $t_c/t_f = a/\sinh (St-St_c)+b$. The critical Stokes numbers for 3D isotropic turbulence are highlighted in bold face.

	2D T-G vortex			2D decaying turbulence			3D isotropic turbulence			
	$W=0$	$W=1$	$W=2$	$W=0$	$W=2$	$W=5$	$W=0$	$W=10$	$W=20$	$W=40$
$St_c$	0.232	0.254	0.328	0.250	0.408	0.575	**0.194**	**0.264**	**0.425**	**0.511**
*a*	0.145	0.149	0.159	0.182	0.188	0.178	0.093	0.093	0.112	0.159
*b*	1.042	1.044	1.053	0.957	0.963	1.030	0.770	0.785	0.793	0.802

### Behavior of particle number density

When the divergence of particle velocity blows up, the particle number density also blows up (Fig. 1d–f). The conservation equation for particle number density $n(\varvec{x},t)$ reads

18 $$\begin{aligned} \frac{\partial n}{\partial t} + \nabla \cdot (\varvec{v}n)=0 , \end{aligned}$$

which can be rewritten as

19 $$\begin{aligned} \frac{1}{n} \left( \frac{\partial n}{\partial t} + (\varvec{v} \cdot \nabla ) n \right) = - \nabla \cdot \varvec{v}=-\sigma _{ii}. \end{aligned}$$

Therefore, the solution for the uniformly distributed initial density $n_0$ can be expressed as

20 $$\begin{aligned} n(t) = n_0 \exp \left[ - \int _0^t \nabla \cdot \varvec{v}(\varvec{r}(t'),t') dt' . \right] \end{aligned}$$

If $\nabla \cdot \varvec{v}$ blows up as $\nabla \cdot \varvec{v} \sim 1/(t-t_c)$, the solution of Eq. (20) can be obtained,

21 $$\begin{aligned} \frac{n(t)}{n_0} \sim \frac{ t_c}{t_c-t}, \end{aligned}$$

or

22 $$\begin{aligned} \frac{n(t)}{n_0} = C_n \frac{ t_c}{t_c-t} , \end{aligned}$$

displaying the behavior similar to that of divergence or $\lambda _D$. Numerical confirmation of this behavior will be given below.

### Behavior of particle vorticity

**Figure 3:**
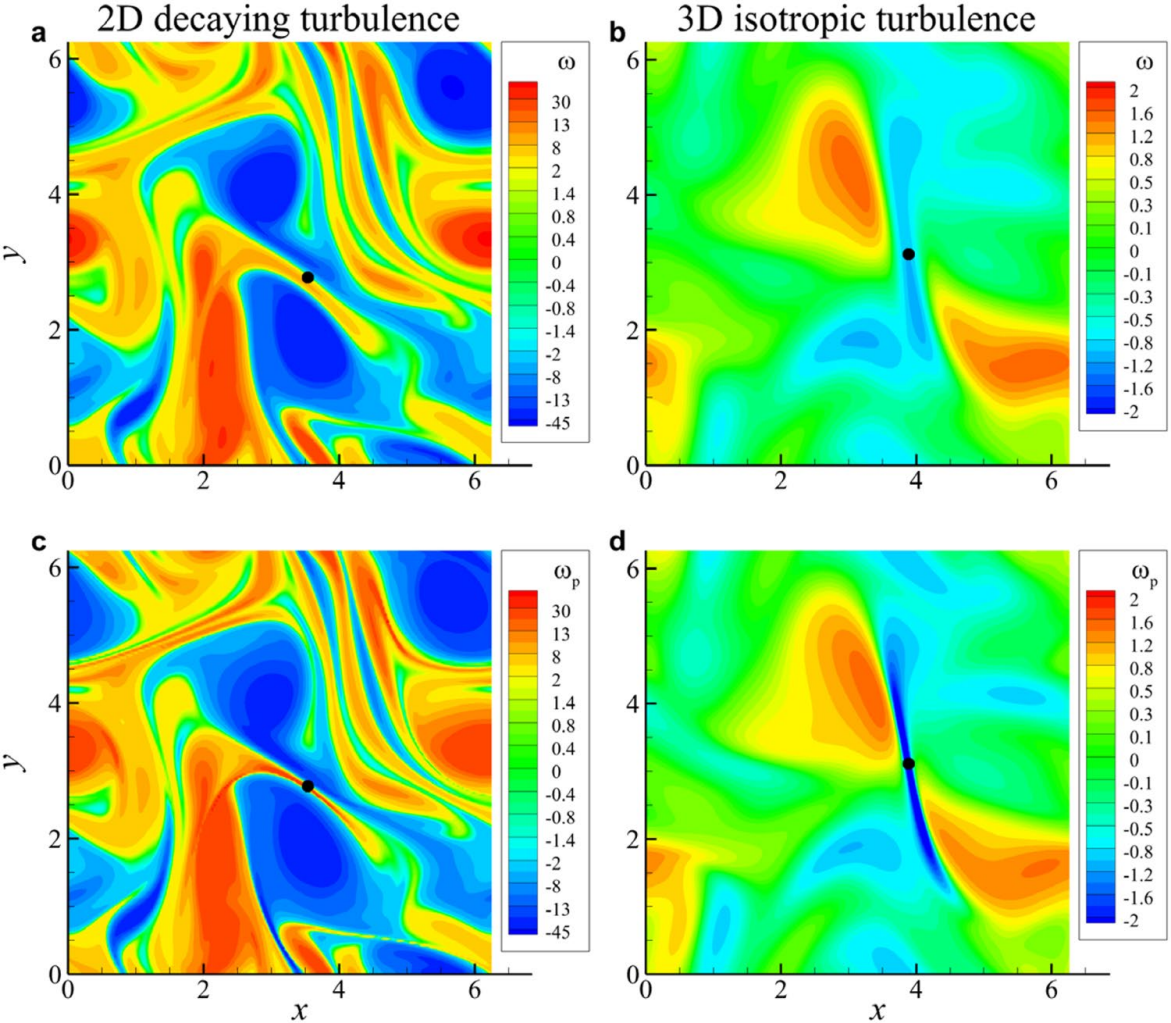
Vorticity fields just before a particle collision. (**a**, **b**) Fluid vorticity fields. (**c**, **d**) Particle vorticity fields. Fluid and particle vorticity fields at the same instant in 2D decaying turbulence (**a**, **c**) and 3D isotropic turbulence (**c**, **d**). The black dot is located where a blowup is going to occur.

If particle velocity is smooth, particle vorticity can be defined by $\varvec{\omega }_p \equiv \nabla \varvec{\times } \varvec{v}$. From now on we discuss the evolution of $\varvec{\omega }_p$ near a blowup. Figure 3 shows the fluid and particle vorticity fields just before a blowup for 2D turbulence and 3D isotropic turbulence. Near the position of a blowup marked by a black dot, fluid vorticity does not show any pronouncing behavior, while particle vorticity appears to be intensified for both 2D turbulence and 3D isotropic turbulence since $\varvec{\omega }_p(\varvec{x},0) = \varvec{\omega }(\varvec{x},0)$ with $\varvec{\omega }$ denoting fluid vorticity. Furthermore, a blowup seems to occur in a thin shear layer formed by two parallel vortices, commonly in 2D turbulence and 3D isotropic turbulence. In 2D Taylor–Green vortex flow, however, no such intensification of particle vorticity was observed since near the blowup, the flow field is irrotational due to the symmetric distribution (Fig. 1).

To understand this growing behavior of particle vorticity, we consider the evolution equation of $\varvec{\omega }_p$ obtained by taking the curl of Eq. (4),

23 $$\begin{aligned} \frac{\partial \varvec{\omega }_p}{\partial t} +(\varvec{v} \cdot \nabla ) \varvec{\omega }_p = (\varvec{\omega }_p \cdot \nabla ) \varvec{v} - \varvec{\omega }_p(\nabla \cdot \varvec{v}) + \frac{\varvec{\omega }-\varvec{\omega }_p}{\tau _p} . \end{aligned}$$

The first and second terms on the right side imply the vortex stretching by straining motion and the vortex intensification by compression, which is usually absent in incompressible fields. Blowup happens in a thin vortex layer (Fig. 3), where converging motion is dominant over stretching motion. Therefore, the vortex stretching term can be neglected near the blowup. Then, multiplying $\varvec{\omega }_p$ to both sides of Eq. (23) yields

24 $$\begin{aligned} \frac{1}{2}\frac{D }{D t}\left| \varvec{\omega }_p\right| ^2 = - \left| \varvec{\omega }_p\right| ^2 (\nabla \cdot \varvec{v}) + \frac{\varvec{\omega }_p\cdot \varvec{\omega }-\left| \varvec{\omega }_p\right| ^2}{\tau _p}. \end{aligned}$$

Near a blowup $|\nabla \cdot \varvec{v}| \gg \tau _p^{-1}$ and thus the last term on the right side of Eq. (24) is negligible. Then, for $\nabla \cdot \varvec{v} \sim 1/(t-t_c)$, the solution to Eq. (24) can be easily obtained,

25 $$\begin{aligned} \omega _p=\left| \varvec{\omega }_p\right| = \frac{C_\omega }{t_c-t} \end{aligned}$$

where $C_{\omega }$ is a constant. This blowup behavior of $\omega _p$ is similar to that of $\nabla \cdot \varvec{v}$ and *n*. Indeed, $\lambda _D, n$ and $\omega _p$ show similar finite-time blowups with a proper constant (Eqs. 17, 22, 25). Numerically estimated constants for the three kinds of flow are listed for $St=0.6, 1$ and 2 and various values of *W* in Table 2. All constants are found to be of order 1 for the range of *St* and *W* considered. Only $C_\lambda$ for 3D isotropic turbulence is around 2.5, which shows a little excursion from the values for 2D Taylor–Green flow and 2D decaying turbulence, while other constants appear to be universal over the three kinds of flow. In particular, all constants for 3D isotropic turbulence show strong universality over the ranges of *St* and *W*.

**Table 2:** Constants of the limiting behavior of $\lambda _D, n$ and $\omega _p$ for various *St* and *W* in three kinds of flow. Only one *W* is applied for each dimension.

St	*W*		$C_\lambda$			$C_n$			$C_\omega$	
	2D	3D	2D T-G	2D DT	3D IT	2D T-G	2D DT	3D IT	2D DT	3D IT
0.6	0	0	1.05	1.32	2.43	0.76	0.77	0.68	0.83	1.20
0.6	1	10	1.09	1.52	2.60	0.79	1.39	0.70	1.35	0.91
0.6	2	20	1.13	1.49	3.70	1.04	1.51	0.80	3.16	0.89
1.0	0	0	1.04	1.16	2.10	0.63	0.65	0.63	0.72	0.92
1.0	1	10	1.05	1.18	2.18	0.65	0.74	0.65	0.68	0.70
1.0	2	20	1.08	1.25	2.39	0.71	0.79	0.69	0.96	0.68
1.0	5	40	1.09	1.20	2.56	1.81	0.85	0.76	0.52	0.72
2.0	0	0	1.01	1.09	2.01	0.56	0.66	0.60	0.57	0.79
2.0	2	20	1.03	1.12	2.05	0.59	0.68	0.63	0.68	0.65

**Figure 4:**
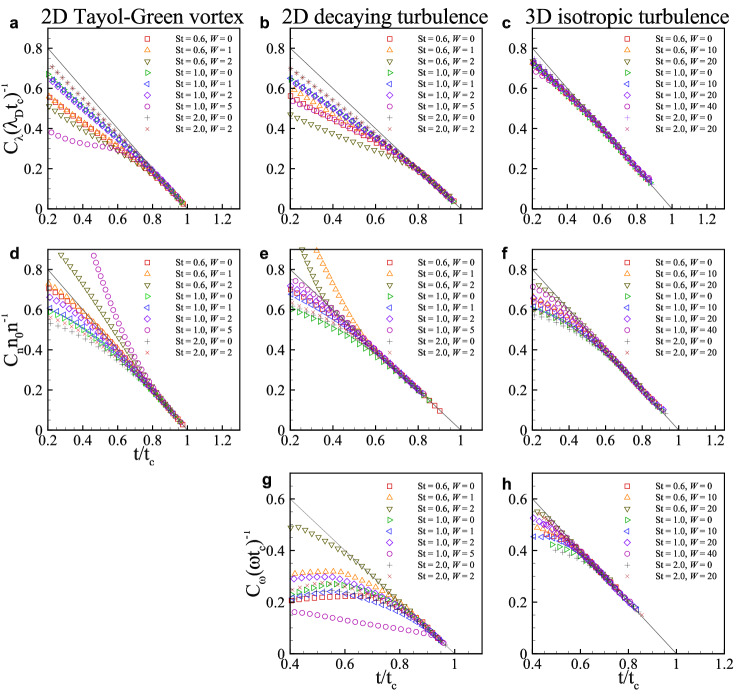
Numerical validation of the blowup behavior of $\lambda _D, n$ and $\omega _p$. (**a**, **d**) 2D Taylor–Green vortex flow. (**b**, **e**, **g**) 2D decaying turbulence. (**c**, **f**, **h**) 3D isotropic turbulence. The solid line denotes $1-t/t_c$.

Equations (17), (22), and (25) are rearranged for easy numerical validation of the blowup behavior,

26 $$\begin{aligned} \frac{C_\lambda }{|\lambda _D| t_c} = \frac{C_n n_0}{n} = \frac{C_\omega }{\omega _p t_c} = 1-\frac{t}{t_c}, \end{aligned}$$

demonstrating a universal relation, for which numerical validation for $St=0.6, 1$ and 2 and for various values of *W* is provided in Fig. 4. This clearly confirms the near-blowup behavior of those variables for the three kinds of flow. Even with the relatively limited resolution for 3D isotropic turbulence, the blowup behavior is well captured although the behavior very near $t_c$ is untraceable further.

### Condition for particle collision in 3D turbulence

**Figure 5:**
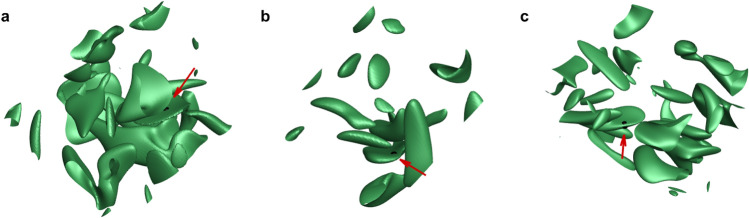
Isosurfaces of $\left| {\varvec{\omega }} \right|$ in three different 3D isotropic turbulence (**a**–**c**). A black dot denotes the location where a blowup or particle collision is going to occur for most Stokes numbers greater than the critical Stokes number at $W=0$. Commonly, a thin vortex layer was found near a blowup.

Since a finite-time blowup implies a collision between particles, we can investigate the flow condition for which a collision between particles could occur. As we confirmed that a blowup occurs in a thin vortex layer of fluid and particle from Fig. 3, we examine more examples for universality of this scenario of collision. Since particle vorticity is usually unavailable, fluid vorticity distribution near a blowup for three different example 3D isotropic turbulence is provided in terms of the magnitude of vorticity in Fig. 5. A black dot indicates the location where it is on the verge of a blowup in each flow. It can be confirmed that a blowup or collision is likely to be occur in a pancake-type thin flat vortex structure induced by nearby vortical structures. It definitely serves as a qualitative condition for a collision.

For a quantitative condition for a collision, we investigate the invariants of the fluid velocity-gradient tensor^37,38^. The fluid velocity gradient $s_{ij} (=\partial u_i/\partial x_j)$ can be decomposed into

27 $$\begin{aligned} s_{ij} = s_{ij}^S + s_{ij}^A , \end{aligned}$$

with

28 $$\begin{aligned} s_{ij}^S = \frac{1}{2} \left( \frac{\partial u_i}{\partial x_j} +\frac{\partial u_j}{\partial x_i} \right) ,~~ s_{ij}^A = \frac{1}{2} \left( \frac{\partial u_i}{\partial x_j} -\frac{\partial u_j}{\partial x_i} \right) , \end{aligned}$$

where $s_{ij}^S$ and $s_{ij}^A$ are the symmetric and the skew-symmetric parts of $s_{ij}$, implying the strain-rate tensor and the rotation tensor, respectively. The eigenvalues $\lambda ^S$ of $s_{ij}^S$, all real, satisfy the following characteristic equation,

29 $$\begin{aligned} (\lambda ^S)^3 + P_S (\lambda ^S)^2 + Q_S \lambda ^S + R_S = 0 , \end{aligned}$$

where $P_S, Q_S$ and $R_S$ are the first, second and third tensor invariants, respectively, given by

30 $$\begin{aligned} P_S= & {} - s_{ii}^S = -(\lambda _1^S+\lambda _2^S+\lambda _3^S) = 0 \end{aligned}$$

31 $$\begin{aligned} Q_S= & {} -\frac{1}{2} s_{ij}^S s_{ji}^S = \lambda _1^S \lambda _2^S + \lambda _2^S \lambda _3^S + \lambda _3^S \lambda _1^S \end{aligned}$$

32 $$\begin{aligned} R_S= & {} -\frac{1}{3} s_{ij}^S s_{jk}^S s_{ki}^S = -\lambda _1^S \lambda _2^S \lambda _3^S \end{aligned}$$

with

33 $$\begin{aligned} \lambda _1^S \ge \lambda _2^S \ge \lambda _3^S, \end{aligned}$$

where Eq. (30) is owing to the incompressibility of fluid and $-4\nu Q_S=\epsilon$, local dissipation rate. For $s_{ij}^A$, a similar characteristic equation holds with the corresponding invariants $P_A, Q_A$ and $R_A$, but $P_A=R_A=0$ due to the skew-symmetricity of $s_{ij}^A$ and

34 $$\begin{aligned} Q_A = -\frac{1}{2} s_{ij}^A s_{ji}^A = \varvec{\omega } \cdot \varvec{\omega } \end{aligned}$$

which is positive definite. Therefore, $\lambda ^A = 0, \pm i |\varvec{\omega }|$.

**Figure 6:**
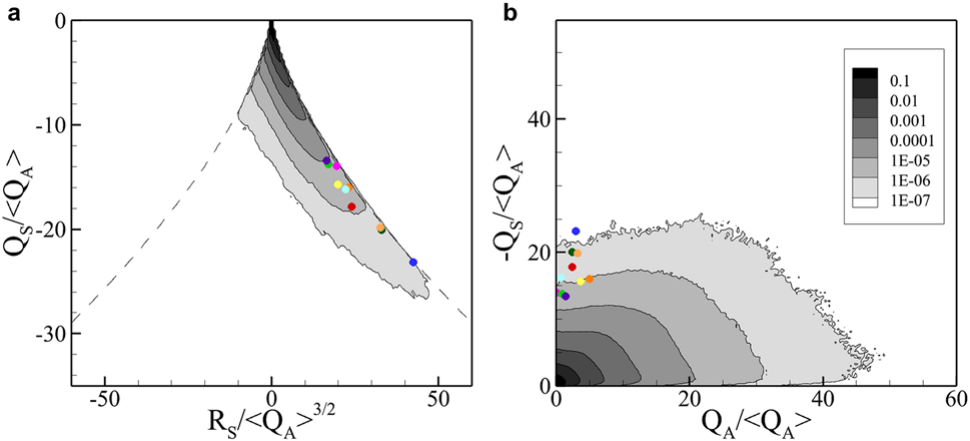
Joint PDF of (**a**) $\varvec{R_S}$ vs. $\varvec{Q_S}$, (**b**) $\varvec{Q_A}$ vs. $\varvec{-Q_S}$. All quantities are normalized by $\langle Q_{A}\rangle$, which is the averaged squared magnitude of the fluid vorticity. Colored dots indicate collision events for $St=1$ and $W=0$ observed in 10 different 3D isotropic turbulent flows.

Distributions of joint probability density functions (PDF) between $R_S$ and $Q_S$, and $Q_A$ and $-Q_S$ for 3D isotropic turbulence are demonstrated in Fig. 6 in which collision events observed in 10 different 3D isotropic turbulences are marked in colored dot. Two observations can be made: First, a collision tends to occur in the place of locally high strain but relatively low rotation, which is rarely found as shown in Fig. 6b. Second, most collision events are found closely to the right branch of the dashed line in the joint PDF between $R_S$ and $Q_S$ (Fig. 6a). The discriminant of the characteristic equation (Eq. 29), $(27/4)R_S^2+Q_S^3$, vanishes on both branches of the dashed line and $sign (\lambda _2^S) = sign (R_S)$. Therefore, on the right branch of the dashed line,

35 $$\begin{aligned} \lambda _1^S = \lambda _2^S = -\frac{1}{2} \lambda _3^S > 0, \end{aligned}$$

implying a straining field with one compressive direction and two equally stretching directions. All of these clearly suggest that a collision preferably occurs in a pancake-like vortex undergoing strong flattening.

## Discussion

To investigate the near-collision behavior of particles in particle-laden turbulence, we studied an Eulerian form of equation of motion of particles (Eq. 4), which was derived under the assumption that the particle velocity is a smooth function of space. Using high-resolution simulations of the 2D Taylor–Green vortex flow, 2D decaying turbulence and 3D isotropic turbulence, we could accurately track a path leading to a blowup of the gradient of particle velocity, which was identified as a signature of particle collision.

The temporal behavior of $\sim 1/(t_c-t)$ predicted to occur in the gradient of particle velocity from an order of magnitude analysis by ref.^1^ was found to hold in the divergence of particle velocity and particularly the most negative eigenvalue of the particle velocity gradient tensor when Stokes number of a particle is greater than the critical Stokes number which was also identified from our investigation. From a parametric study with Stokes number and gravity factor for the three different kinds of flow, we observed that as Stokes number increases, the blowup or collision occurs earlier and strong gravity delays the blowup.

When a blowup occurs, along with the divergence of particle velocity, particle number density also blows up in the similar manner, commonly for all three kinds of flow. Although our Eulerian simulation of particle motion is valid until the first blowup occurs somewhere, this direct calculation of particle number density could be very useful for multifractal modeling of uneven distribution of particles^10,20,21,39^.

Particle vorticity, which means spin velocity of a particle, exhibits similar blowup in both 2D decaying turbulence and 3D isotropic turbulence when the divergence of particle velocity blows up. From the equation for particle vorticity and simulation results, we identified that particle vorticity blows up through vortex intensification owing to compressive field of particle velocity. This is different from the finite-time blowup of vorticity of the solution to Euler equation through vortex stretching, which is possible only in 3D or axisymmetric flows^40–48^. It is interesting to notice from the equation for particle vorticity (Eq. 23) that the blowup due to compressive motion is suppressed in the incompressible Euler equation by pressure field, enforcing the divergence-free velocity field. In the particle motion, the Stokes drag force plays a similar role to pressure when Stokes number is below the critical Stokes number.

Finally, we investigated the flow condition most probable for particle collision in 3D isotropic turbulence. From various cases, we noticed that a thin flat vortical structure is the location where a particle collision is most likely to happen (Fig. 5). From a more detailed investigation of the invariants of the fluid velocity-gradient tensor, we confirmed that a collision preferably occurs in a pancake-like vortex under the compressive strain.

Our results of the near-collision behavior of particles are in principle valid for infinitesimally small particles. When the size of a particle is finite, however, the effect of size such as the lubrication forces between colliding two particles should be considered in the study of the near-collision behavior. Several collision models accounting for the lubrication forces before collision and the contact forces during collision have been proposed from particle-resolved simulations based on the immersed boundary method^49–53^. However, given that the lubrication forces are non-negligible only when the gap between the colliding particles is much less than the radius of a particle, the near-collision behavior found in our study remains valid until the contact of two particles occurs as long as the particle size is much smaller than the flow length scale such as the Kolmogorov length scale.

We demonstrated from an Eulerian investigation of particle velocity that a blowup leading to particle collision can be accurately identified. This approach can be extended to an investigation of fractal structure of bubble motion in turbulence the behavior of which is different from that of heavy particles in that small bubbles hardly collide with each other due to small Stokes number.

**Figure 7:**
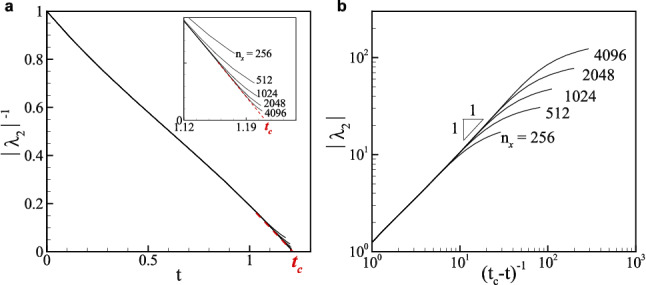
Identification of a finite-time blowup in the two-dimensional Taylor–Green vortex flow for various numerical resolutions. $\lambda _2$ is the most negative eigenvalue of $\sigma _{ij}^S$ that is the symmetric part of the particle velocity gradient for $St=1$ and $W=0$. (**a**) Inverse of $|\lambda _2|$ in time. (**b**) $|\lambda _2|$ in $(t_c-t)^{-1}$. The critical time $t_c$ is estimated from the behavior of $1/|\lambda _2|$ near the blowup using $|\lambda _2| \sim (t_c-t)^{-1}$ as described in (**a**).

## Methods

### Direct Eulerian simulations of particle-laden flows

Equations (4), (10), (11) and (12) are solved by a spectral method to maintain the spectral accuracy, and a time advancement is accomplished using a third-order Runge-Kutta method. Spatial resolution is kept at $4096^2$ for the Taylor–Green vortex and 2D decaying turbulence and $256^3$ for 3D isotropic turbulence. For the identification of the flow condition for a collision, an investigation of the invariants of the fluid velocity-gradient tensor was carried out using $128^3$ resolution for 3D isotropic turbulence at $Re_\lambda = 70$. The Eulerian particle equation is solved in the frame moving at the settling velocity to avoid accumulation of numerical errors. In all cases, the initial condition for the particle velocity is $\varvec{v}(\varvec{x},0)=\varvec{u}(\varvec{x},0)+\varvec{w}$, which is smooth in space. The main nondimensional parameters are the Stokes number defined by $St\equiv \tau _p/\tau _f$ and the normalized settling velocity $W\equiv |\varvec{w}|/v_f$, where $\tau _f$ and $v_f$ are the flow time and velocity scales, respectively. For the 2D Taylor–Green vortex and 2D decaying turbulence, $\tau _f=1/\omega _{rms}$ and $v_f=u_{rms}$ where $\omega _{rms}$ and $u_{rms}$ are root-mean-square vorticity and velocity of fluid, respectively. For the 3D isotropic turbulence, $\tau _f=\tau _\eta$ and $v_f=\eta /\tau _\eta$.

### Condition for resolution

To identify a blowup, we track large amplitude anomaly of the gradient of particle velocity, particularly the most negative eigenvalue of the symmetric part of the particle velocity gradient tensor since a blowup would occur when particles accumulate instead of creating vacuum. Figure 7 shows an example of a blowup in the 2D Taylor–Green vortex, in which the most negative eigenvalue $\lambda _2$ blows up in finite time. It clearly indicates $1/(t-t_c)$ behavior as $t \rightarrow t_c$ (Fig. 7b) and as the resolution increases such behavior is better captured near $t_c$ (inset of Fig. 7a). Extrapolation of the linearly decreasing behavior of $1/|\lambda _2|$ near $t_c$ also determines $t_c$ (Fig. 7a). Although an adoption of an adaptive mesh would yield better numerical evidence for a blowup^45,48^, the current resolution seems to be sufficient for the confirmation of the asymptotic scaling behavior as demonstrated in Fig. 4.

## Data Availability

The data supporting the findings of this study are available from the corresponding author upon request.

## Code availability

The simulation codes that have been used to produce the results of this study are available from the corresponding author upon request.
